# Supplementary figures and images for: Increased Efficacy of Histone Methyltransferase G9a Inhibitors Against MYCN-Amplified Neuroblastoma
Source: Front Oncol. 2020 May 27;10:818. doi: 10.3389/fonc.2020.00818 (PMC7269128; doi:10.3389/fonc.2020.00818)

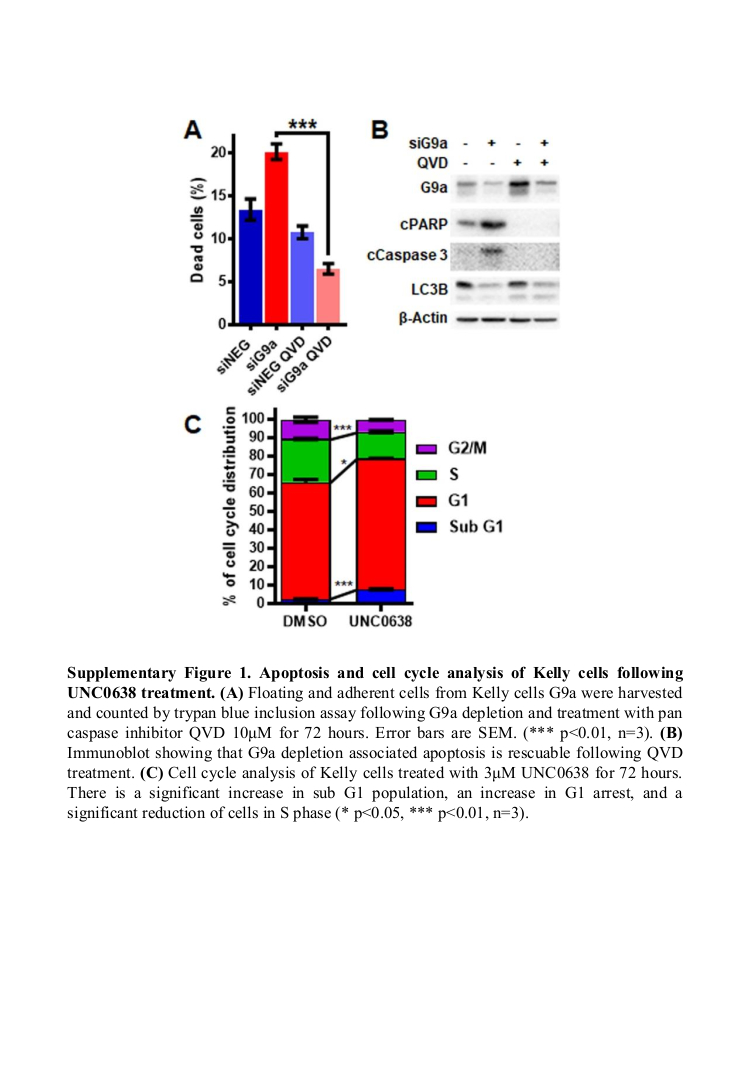

Supplement: Supplementary file 1 [file Image_1.JPEG]

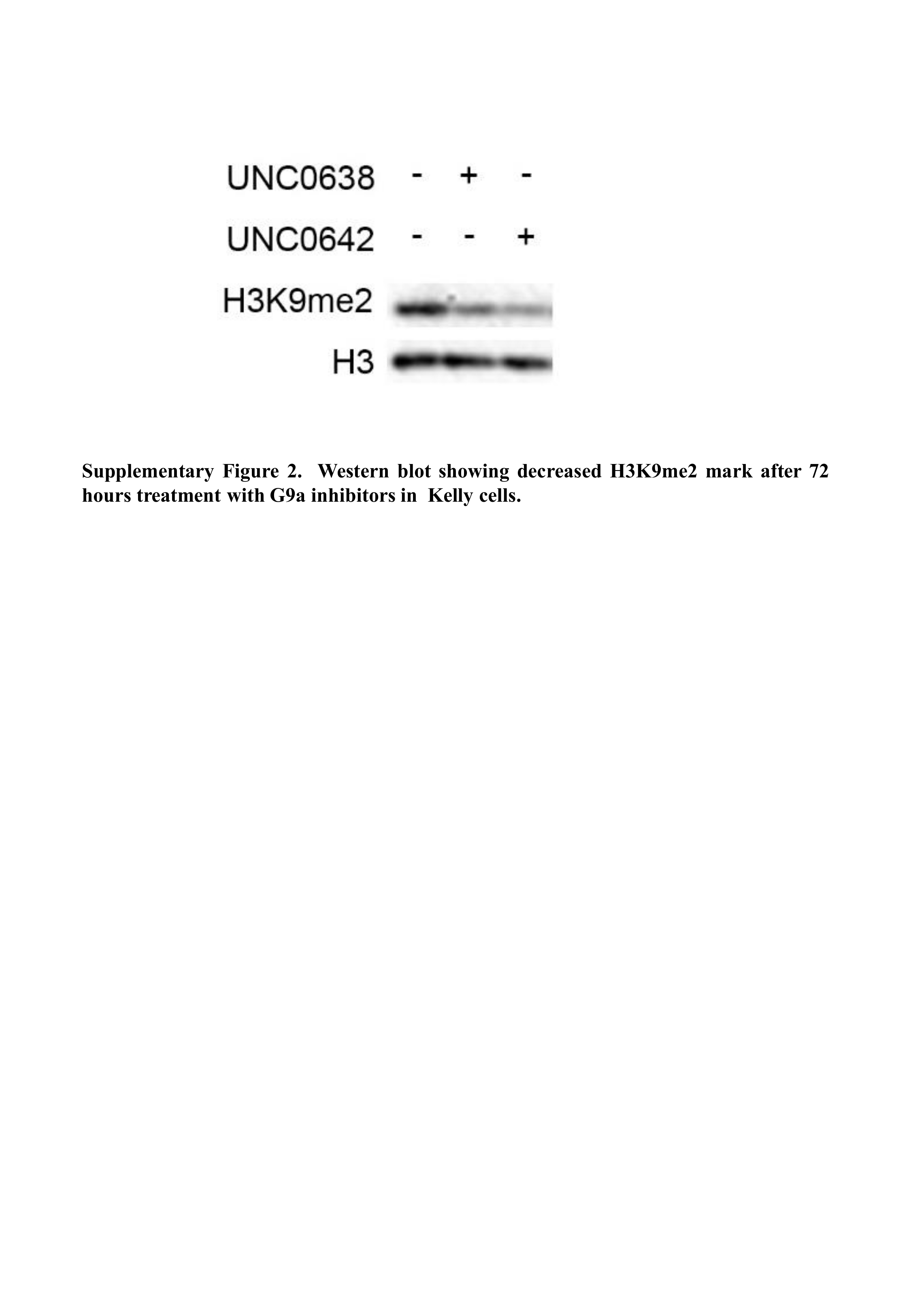

Supplement: Supplementary file 2 [file Image_2.JPEG]
